# MATE: Machine Learning for Adaptive Calibration Template Detection

**DOI:** 10.3390/s16111858

**Published:** 2016-11-04

**Authors:** Simon Donné, Jonas De Vylder, Bart Goossens, Wilfried Philips

**Affiliations:** iMinds - IPI, Ghent University, Ghent B-9000, Belgium; Jonas.DeVylder@telin.ugent.be (J.D.V.); Bart.Goossens@telin.ugent.be (B.G.); philips@telin.ugent.be (W.P.)

**Keywords:** computer vision, camera calibration, checkerboard detection, deep learning

## Abstract

The problem of camera calibration is two-fold. On the one hand, the parameters are estimated from known correspondences between the captured image and the real world. On the other, these correspondences themselves—typically in the form of chessboard corners—need to be found. Many distinct approaches for this feature template extraction are available, often of large computational and/or implementational complexity. We exploit the generalized nature of deep learning networks to detect checkerboard corners: our proposed method is a convolutional neural network (CNN) trained on a large set of example chessboard images, which generalizes several existing solutions. The network is trained explicitly against noisy inputs, as well as inputs with large degrees of lens distortion. The trained network that we evaluate is as accurate as existing techniques while offering improved execution time and increased adaptability to specific situations with little effort. The proposed method is not only robust against the types of degradation present in the training set (lens distortions, and large amounts of sensor noise), but also to perspective deformations, e.g., resulting from multi-camera set-ups.

## 1. Introduction

Perspective cameras are typically modeled as pinhole cameras with some additional lens distortion [1]. Under this model, projection is separated in an extrinsic matrix (location and orientation of the camera), an intrinsic matrix (focal distance, skew and optical center) and the deformation coefficients (typically the Brown–Conrady ”plumb bob” distortion model [2]). Camera calibration comprises the estimation of the intrinsic matrix and the deformation coefficients, both of which are camera-dependent but remain constant in different scenes.

**Figure 1:**
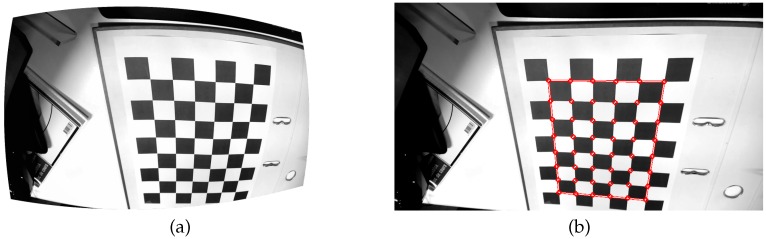
Example of the detection of a checkerboard. A radially distorted input image (**a**) and the undistorted image with the detected points highlighted in red (**b**).

To estimate the camera-specific parameters, calibration objects are used: physical objects with a known 3D model. By observing the projection of the calibration object, the lens deformation and intrinsic parameters can be estimated [3,4,5,6,7]. The calibration template used is typically a monochrome checkerboard, of which we need to detect the corners as illustrated in Figure 1. In this paper, we outline a checkerboard corner detection method based on a deep convolutional net. The training aspect of the network means that it can be deployed as a generally applicable detection method, but that it can be tailored to specific problems or scenarios as well. We test two instances of the network: one is trained using distorted input images with nearly no noise, while the other is also trained on noisy images. We illustrate adaptivity by training the network specifically for a special hexagonal color-based calibration template as well.

### Existing Methods

Several techniques exist for the detection of checkerboard features, as well as for constructing checkerboards from them. Initial approaches used general feature detectors such as the Harris [8], SUSAN (Smallest Univalue Segment Assimilating Nucleus) [9,10] or Moravec [11] features. Because of the distinct nature of the checkerboard template, it is possible to do a preliminary filtering for areas of the image likely to contain a checkerboard [12,13]. A more complex combination of general image features is proposed by Placht et al. with ROCHADE (Robust Checkerboard Advanced Detection) [14]: the centerlines of a thresholded Scharr-filtering of the input image are calculated and used to compute the saddle-points, which are the detected corners. After subpixel refinement, the checkerboards are constructed.

In the open-source computer vision library OpenCV, the checkerboard detection method is an algorithm by Vezhnevets [15], which operates by detecting black quadrangles in the image and combining those into checkerboards. This approach was extended in OCamCalib [16,17] with a better checkerboard construction algorithm and pre-processing to handle blurred or distorted images.

As observed in [18], the checkerboard can be detected on the edge image as two sets of lines converging on two separate vanishing points. They can be detected successfully using the Hough transform [18,19] as long as the lens distortion of the camera keeps the lines straight. For wide-angle cameras (such as the GoPro series), this assumption does not hold and such methods are not applicable. This would not be an issue if we could correct for the lens distortion, which requires knowledge of the camera’s intrinsic parameters—the very reason we are trying to match the checkerboard pattern. To escape this catch-22 situation, the template extraction algorithm should be robust against such lens distortions.

**Figure 2:**
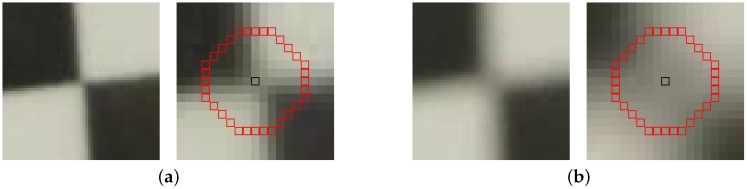
Illustration of the effect of focal blur on the corners. (**a**) in-focus corner, with the six-radius circular boundary highlighted on a detailed version; (**b**) badly out-of-focus corner with the similar enlarged version.

Recently, more specific features have been proposed specifically designed for checkerboard feature detection [10,12,20,21,22]. In [20,21,22], circular neighborhoods of the corner candidates are considered (see also Figure 2): the intensities of the circular boundary form the corner candidate’s feature vector. By counting and scoring the sign changes of this circular boundary, the authors of [21,22] are able to select the corner points of the checkerboards: checkerboard corners sport four distinct intensity steps over the feature vector, or a characteristic path. With ChESS (Chessboard Extraction by Subtraction and Summation) [20], it is shown that while such an approach works well, it may result in many false positives, and a more complex criterion is formulated based on the circular boundaries and the correlations of its phase-shifted versions. Bennett et al. go on to discuss the various false positives and extend their ChESS feature to account for these [20]. The main drawback is that the technique has been tailored for a low degree of lens distortion: the detection assumes orthogonal angles in the checkerboard quadrangles—a valid assumption when the checkerboard is parallel to the imaging plane, but less practical in a multi-camera set-up. In [10,12], the local neighborhood of corner candidates is considered, rather than only a circular boundary. Zhu et al. [10] match circular corner templates to the local neighbourhoods, while Arca et al. [12] divide the neighborhood into nine sectors: a center and eight sectors. The statistics of those nine sectors are compared against hard-coded rules for corner detection.

There have been some forays into the field of machine learning for feature detection. Notably, the FAST (Features from Accelerated Segment Test) [23] image corner detector is built upon machine learning foundations, as is its successor, FAST-ER (FAST - Enhanced Repeatability) [24]. Deep learning has proven effective in e.g., the segmentation of electron-microscopy images [25], MRI images [26] and hyperspectral images [27]. In a broader scope, it has recently also proven effective at pixel-wise processing: the authors of [28] train a deep convolutional net for image super-resolution, which consists of just three layers.

It illustrates that even a few well-trained layers can be extremely effective for pixel-wise processing. However, the main body of machine learning and deep learning literature has focused on high-level image features, such as those required for classification of images in ImageNet [29]) or detection of handwritten digits in the MNIST (Mixed National Institute of Standards and Technology) dataset [30]. Schmidhuber [31] has compiled an overview of deep learning techniques and their history, and we refer interested readers to that compendium.

Early attempts explored the possibility to use neural networks for camera calibration [32,33,34]. Memon and Khan [32] actually circumvent camera calibration: focusing on a specific stereo set-up, they pre-train a neural network to perform the conversion from 2D to 3D locations; the major drawback being that this method needs to be retrained for every different stereo set-up. Jun and Kim [33] use a similar approach not limited to a stereo scenario; they also propose two different multi-layer perceptrons: one for the center area of the image plane, and one for the outer area that has larger radial distortion. Ahmed et al. [34] proposed a feed-forward network to perform the inverse function: their neural network transforms a 3D location into a 2D location in the image domain, thus modeling the camera parameters implicitly.

## 2. Proposed Approach

**Figure 3:**
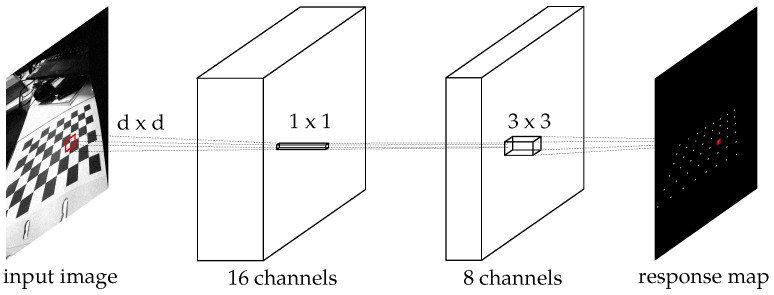
Overview of the proposed machine layers. The input image is first filtered with a relatively large kernel size (proportional to image content) into 16 feature channels. A direct filtering (no spatial influences) into eight new channels. The last step achieves a single-channel response map. The activation functions at each step are ReLUs (rectified linear units [36]).

We propose a convolutional net for the detection of the checkerboard corners. This network can be trained against a general dataset, as well as tailored towards application-specific scenarios. The proposed network consists of three layers: the first is intended to extract a series of features from pixel neighborhoods, while the final two will combine these features into a meaningful chessboard corner score. Figure 3 gives an overview of the machine we propose.

The first layer of the network consists of a relatively large kernel size convolutional filter with many output channels. Its activation function is a ReLU (Rectified Linear Unit) [35]. This first filter is given a large spatial radius because of the good results obtained by circular boundaries [20,21]. The radius of the spatial filter at this stage should be large enough to overcome the effects of the image blur on the corners, as illustrated in Figure 2. This blur is typically the result of a badly configured focal distance at acquisition time. As most if not all existing cameras carry an built-in autofocus module, this effect is typically small. We have chosen a radius of six pixels, which is more than sufficient for the scenarios we evaluate—it is slightly larger than the radius in [20]. Even larger spatial radii would allow for more focal blur: in most cases, a larger radius is superfluous while slowing down the processing; we will explore this trade-off in the results section. Finally, we assume that the input images are gray-scale.

We denote the input image as X. The first layer results in 16 channels $L_{1,i}\left(X\right)$, governed by trained filter kernels $W_{1,i}$ and bias $b_{1,i}$:

(1) $$L_{1,i}\left(X\right)\left(x,y\right)=\max\left(\left(W_{1,i}\times X\right)\left(x,y\right)+b_{1,i},0\right).\;\forall i=1\ldots 16.$$

Earlier, the spatial support d×d of the kernels $W_{1,i}$ should be large enough to cover the corner size in the images, subject to the focal blur. We have chosen 13×13, which is shown in the results section to be large enough.

The next layer is a local translation of the 16 feature channels into eight new features, each with another ReLU activation function: ReLU(x)=max(x,0). There is no spatial influence at this layer, and it is solely meant to combine the local neighborhood characteristics into meaningful higher-level features by exploiting the non-linearity of the activation functions. Each of the eight output channels is a weighted sum of the 16 input channels with biases $b_{2,j}$:

(2) $$L_{2,j}\left(X\right)\left(x,y\right)=\max\left(\sum_{i=1}^{16}a_{i,j}\left[L_{1,i}\left(X\right)\right]\left(x,y\right)+b_{2,j},0\right).\;\forall j=1\ldots 8.$$

The last layer combines the eight channels resulting from the second layer into a single response map. This layer sports a small spatial support to allow for neighborhood influences in the response map. Similar to earlier layers, the output of this layer is given by:

(3) $$L_{3}\left(X\right)\left(x,y\right)=\max\left(\sum_{j=1}^{8}\left(W_{3,j}\times \left[L_{2,j}\left(X\right)\right]\right)\left(x,y\right)+b_{3},0\right).$$

The number of parameters in a layer is given by $C_{\mathrm{out}}\left(C_{\mathrm{in}}\times d_{\mathrm{layer}}^{2}+1\right)$, where $C_{\mathrm{in}}$ and $C_{\mathrm{out}}$ denote, respectively, the number of input and output channels, and the spatial support of the kernel is given by $d_{\mathrm{layer}}$. In our proposed approach, this equates to $16\left(Cd^{2}+1\right)+8\left(16+1\right)+\left(8\times 3^{2}+1\right)$. For gray-scale inputs and a spatial support of 13×13 for the first layer mentioned earlier, there are 2929 parameters to train. On the other hand, the cardinality of the training input is large enough to bypass this. Consider that a 640×480 gray-scale image contains effectively 291,716 input samples (discounting the border pixels), each of which has a linked ground-truth response value. Although these input samples overlap to a large degree, this overlap is required: while the locations of the checkerboard corners should receive a large response, their close neighborhood should fetch much lower responses (i.e., the black–white edges). Of those input samples, only 48 are true positives (at least in our training set, this depends on the dimensions of the calibration template)—we explain how to handle this discrepancy in Section 3.1.

The first layer’s Equation (1) has assumed that the input image contains only a single channel: this is enough for the detection of the characteristic monochrome checkerboard. If specific applications call for *C* input channels $X_{c}$ (C>1), the first layer formulation can be rewritten to be similar to the third layer:

(4) $$L_{1,i}^{\prime }\left(X\right)\left(x,y\right)=\max\left(\sum_{c=1}^{C}\left(W_{1,c,i}\times X_{c}\right)\left(x,y\right)+b_{1,i},0\right).$$

### 2.1. Computational Complexity

The complexity of the proposed approach is only dependent on the size of the spatial support in the first layer. In total, there are 16 of these d×d convolutions, 128 direct translations (convolutions without any spatial support) and eight 3×3 convolutions. Finally, the non-local-maximum suppression and thresholding require only a small number of additional calculations. Keeping in mind that there are also the ReLU activation functions, one additional comparison per pixel per channel is introduced. This makes for a total of $(16d^{2}+191)N$ additions and multiplications (where *N* is the number of pixels in the image), and 25N binary comparisons.

### 2.2. Mapping Existing Approaches on the Proposed Network

The neural net can be interpreted as a generalized formulation of existing solutions. Following the same skeleton, but fleshing it out in an optimal way: through training, we estimate the optimal decision rules, as far as the training set is concerned. Assuming that the training set is representative for the actual input images, the trained neural net is applicable to unseen inputs: we make sure that the training set contains plenty of examples of, e.g., noisy and distorted inputs, to make the resulting network robust to these degradations.

For example, the approach from [20] can be directly mapped onto the proposed network. Let the first layer implement the various terms in the so-called sum- and difference-responses from that work, as well as the neighbor and local mean. The second layer then combines these characteristics into the sum-, difference- and mean-responses defined in [20]. Because of the absolute values involved, we need twice the intermediate channels to let the ReLU create the absolute value. The final layer combines the various responses into a single response map for decision making. With similar reasonings, the methods of [10,12,21,22] can be approximated by the proposed network.

## 3. Experiments and Results

We first discuss the training of the network, after which we evaluate the trained network on datasets from literature. The proposed neural net is used to detect corner candidates, which can be refined using sub-pixel precision approaches such as those from [14,37]. Therefore, the evaluation consists of counting the number of type I and type II errors (false positives and false negatives): a detection is assumed to be correct if it lies close to a ground-truth location.

### 3.1. Training the Network

The network is trained by the use of stochastic gradient descent [38] (SGD). To speed up the iterations, and hence convergence, SGD uses a subset of the entire training set at each update step. A common practice is to simultaneously back-propagate as many samples as possible, restricted by memory and computational constraints. In addition, a momentum term is used to mitigate the effect of local optima in the parameter space [39,40]. The dataset for training consists of two parts: images as captured by us directly (using a Logitech C930e Pro (Logitech, Romanel-sur-Morges, Switserland), at full HD resolution) and digitally altered versions of these captured images.

**Figure 4:**
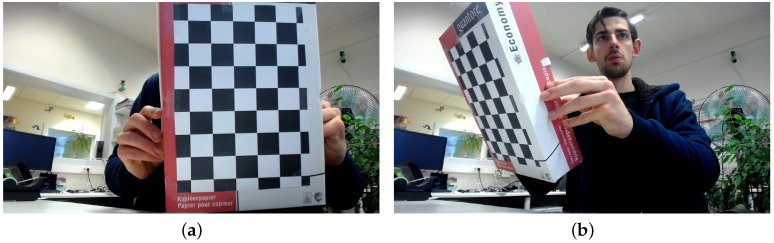
Two examples from the captured training data set, ranging from clean front-view images (**a**) to oblique vantage points (**b**).

The captured image set $P_{\mathrm{captured}}$ (a total of 85 input images) cover a wide range of board orientations as illustrated in Figure 4, resized to half their original resolution (960×540). The background of the captures was intentionally kept cluttered, as might be the case in actual calibration captures. The camera used in these captures has little lens distortion and capture conditions were good, generally speaking. The first set of digitally altered versions, $P_{\mathrm{rotated}}$, consists of (90,180,270)-degree rotations of these input images, half of which have their intensities inverted (so that white becomes black and vice versa). This already increases the training set fourfold, as well as forcing the detector to become rotation and (somewhat) intensity invariant in as far as the original dataset did not embody this constraint yet. We call the clean training set $P_{\mathrm{clean}}=P_{\mathrm{captured}}\cup P_{\mathrm{rotated}}$.

**Figure 5:**
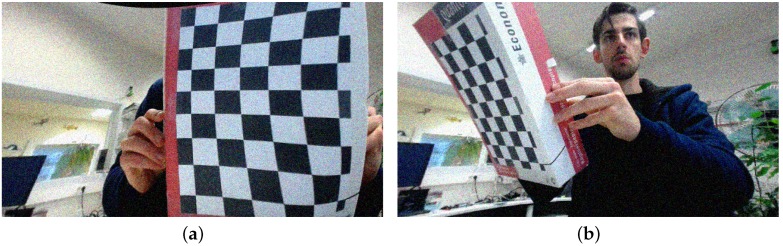
Two examples from the full training data set. These are the corresponding images from Figure 4 with added distortion and noise as discussed in Section 3.1.

Because the distortions and noise in $P_{\mathrm{clean}}$ are relatively low, we artificially add both. We add both radial and tangential distortion [41,42] as well as Gaussian noise to the clean training set to simulate poor image quality. The five radial and tangential distortion parameters are uniformly distributed between 0 and 0.1, while the added Gaussian noise had a standard deviation of 0.1. The resulting noisy and distorted training set $P_{\mathrm{full}}$ is illustrated in Figure 5. These values of distortion range from virtually no distortion to unlikely amounts of distortion as in the left part of Figure 5.

We collect all the parameters of the neural net into a single vector $\vec{p}$ and call the binary ground-truth image of corner locations G(x,y). Ground-truth corner locations are obtained through manual annotations: the four outer corners of the checkerboard are indicated manually, and then the interior corners are interpolated. Finally, all of these points converge locally to the saddle points, after which they are checked manually to correct wrong corners. As the penalization function $\Phi \left(\vec{p}\right)$ for the optimization problem, we use the one-sided quadratic difference:

(5) $$\Phi \left(\vec{p}\right)=\sum_{(x,y)\in \Omega }\left\{\begin{array}{cc} \max{\left(1-L_{3}\left(X\right)\left(x,y\right),0\right)}^{2}, & \mathrm{where}\;G(x,y)=1,\\ \max{\left(L_{3}\left(X\right)\left(x,y\right),0\right)}^{2}, & \mathrm{where}\;G(x,y)=0. \end{array}\right.$$

This cost function penalizes responses of corner locations that are lower than 1, and responses of non-corner locations which are higher than 0—this is similar to the maximal margin from support vector machines. By enforcing a non-zero margin, the binary classification needs to be more discriminative. At testing time, we chose the classification threshold to be 0.5, but this could be adjusted in either direction for more precision (higher values) or more recall (lower values).

Additionally, we include weights for all of the responses. Because of the disparity between the number of ground-truth positives and negatives, we weight the cost in each location by the occurrence of that ground-truth state. Calling the number of positive ground-truths $N_{P}$ and negative ground-truths $N_{N}$, the final cost function becomes

(6) $$\Phi \left(\vec{p}\right)=\sum_{(x,y)\in \Omega }\left\{\begin{array}{cc} \frac{1}{N_{P}}\max{\left(1-L_{3}\left(X\right)\left(x,y\right),0\right)}^{2}, & \mathrm{where}\;G(x,y)=1,\\ \frac{1}{N_{N}}\max{\left(L_{3}\left(X\right)\left(x,y\right),0\right)}^{2}, & \mathrm{where}\;G(x,y)=0. \end{array}\right.$$

We ignore any locations near the borders of the image as well as close to the ground-truth locations: we will select corner candidates by non-maximum suppression on the response map. This means that we can allow points in the immediate neighborhood of corners to have a high response as well: those locations are therefore don’t care. This makes the training phase robust against small ground-truth inaccuracies.

As a side note, the implementation of the neural net and the training were done in Quasar, a programming language which allows for straightforward GPU (Graphics Processing Unit) implementations [43]. This is a large advantage for the processing of images, as the high degree of parallelism and spatial locality of the pixel data can be easily exploited.

### 3.2. Datasets

For the evaluation, we use both the training datasets and two datasets from [14]. Obviously, MATE (Machine learning for Adaptive Template Extraction) trained using $P_{\mathrm{full}}$) and MATE${}^{*}$ (trained using $P_{\mathrm{clean}}$) are assumed to perform well on the training sets, as they were optimized over those explicitly. For a more representative comparison, we also include the performance on two datasets introduced by Placht et al. [14]: uEye and GoPro.

The two training sets, $P_{\mathrm{clean}}$ and $P_{\mathrm{full}}$, were discussed earlier and are illustrated in Figure 4 and Figure 5.

**Figure 6:**
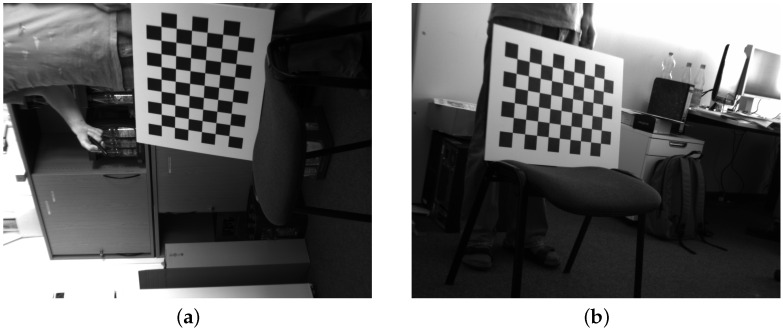
An example from the uEye stereoscopic dataset [14]: the left (**a**) and right (**b**) views.

The uEye dataset is captured by two IDS UI-1241LE cameras (Imaging Development Systems, Obersulm, Germany), in a wide-baseline set-up. The lens distortion is insignificant, and the image resolution is 1280 by 1024: these images are used as-is, as illustrated in Figure 6. This dataset will serve to evaluate the robustness against perspective transforms: the chessboards in the uEye dataset are typically at an angle to the imaging plane because of the wide-baseline set-up. Both MATE${}^{*}$ and MATE are assumed to perform well on this dataset, as both are trained against perspective transforms.

**Figure 7:**
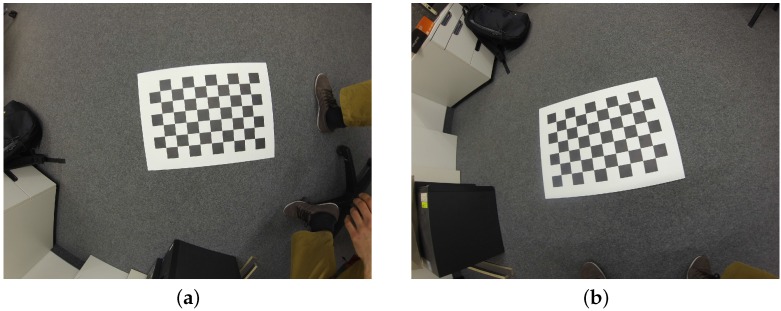
Two examples from the GoPro dataset [14]: the same scene from two vantage points (**a**) and (**b**). Even for the head-on view (**a**), significant warping is present in the image.

The GoPro dataset, on the other hand, has a very large resolution (4000 by 3000) and a generally good quality. However, the wide-angle lens of the GoPro introduces significant lens distortion, and therefore this dataset illustrates the robustness against lens distortions. The images are used at half-resolution: as illustrated in Figure 2, the spatial support required would grow too large for an efficient execution. The goal is to detect the corners; (sub)pixel refinement occurs afterwards at a local level—much less affected by the image resolution. Other methods receive these same down-sampled images displayed in Figure 7 as input.

### 3.3. Evaluation

We perform several comparisons with the state-of-the-art methods ChESS [20], ROCHADE [14] and OCamCalib [17]. We evaluate the methods both on the training datasets we used as well as the uEye and GoPro dataset from [14]. Additionally, we evaluate the methods on an ”angle” dataset, which is used to illustrate the robustness of the methods to checkerboard skew.

**Figure 8:**
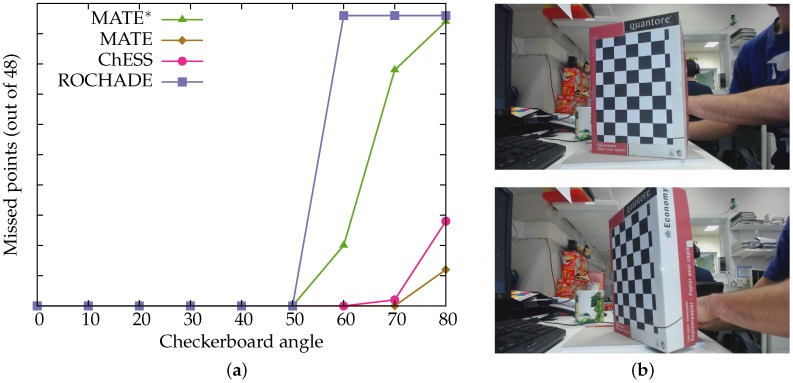
The results from the ’angle’ dataset. (**a**) the checkerboard angle against the number of missed points; (**b**) example images (20 and 60 degrees, respectively).

Results for the training datasets are given in Table 1 and Table 2. The uEye and GoPro dataset results are available in Table 3 and Table 4, and the results from the ”angle” dataset are shown in Figure 8. The two networks trained on, respectively, the clean and full datasets are denoted with MATE${}^{*}$ and MATE. In practice, we use MATE: as shown below, this method sacrifices some precision in favor of recall; the subsequent checkerboard construction algorithms we use have no issue coping with this relatively small increase of false positives.

We evaluated the raw detections, without any sub-pixel refinement steps. Groundtruth was annotated manually and subsequently subject to local convergence to the corner location. Next, detected corner candidates are linked to the closest ground truth corner. If the distance is less than five pixels, this is counted as a true positive. The accuracy gives the average distance between true positives and their ground truths. The missed corner rate and double detection rate denote how many ground truths have either zero or several detections. For the MATE detectors, we perform non-maximum suppression on the neural network output and then apply a threshold of 0.5. The ROCHADE method’s public implementation was used; the ChESS detector was re-implemented in the Quasar language. OCamCalib’s publicly available source code was used in its evaluation. The number of detected checkerboards for this method is self-reported: this means that it may detect all checkerboards even when when some points are missing. Given the nature of the OCamCalib method, it is not meaningful to decouple point detection and checkerboard construction.

We can see from Table 1, Table 2, Table 3 and Table 4 that the trained neural nets do not lose performance over state-of-the-art techniques. While, as expected, they trump on the training datasets, they do not lose performance on an external dataset (Table 3). We notice that the neural net trained on the full training set allows more false positives in return for less false negatives, a result of being trained on noisy and distorted samples. The execution time for ROCHADE remains constant because it rescales large images, while the execution time of OCamCalib varies depending on the difficulty of detecting the checkerboard.

Notably, MATE${}^{*}$ does not lose much accuracy over MATE on the GoPro dataset, even though this dataset contains high degrees of lens distortion—a scenario MATE${}^{*}$ was not explicitly trained for. We conclude that the various types of perspective transforms in MATE${}^{*}$’s training set conferred enough robustness to handle these distortions. The large difference in accuracy between MATE and MATE${}^{*}$ on the full training set is assumed to be the result of the noise, a factor for which MATE${}^{*}$ was woefully unprepared.

In the noise-free datasets, OCamCalib performs the best out of the tested methods, including the proposed method. It detects all of the chessboards. However, it counts a checkerboard as detected even when some points are still missing. There are two main drawbacks to OCamCalib: it requires the dimension of the checkerboard to be known in advance, and it requires the chessboards to have a white border. This is an issue on the training sets, in which half the checkerboard have a black border because of the intensity reversal. For this reason, this method was only run on the non-inverted half of those datasets.

We conclude that the trained neural network is able to match or outperform other, hand-designed, corner-point detectors in all tested scenarios. In noise-free settings, it is hard to beat the performance of OCamCalib, which uses higher-level info (the checkerboard size and inter-point relations) to detect and build the checkerboard. However, in the noisy dataset included here, OCamCalib misses the detection of a large number of points, which the proposed method as well as ChESS [20] do detect.

Figure 8 shows how the various methods cope with the detection of a chessboard under various angles. The neural net trained on the full training set is able to detect the full chessboard under a 70-degree angle, while the other methods lose the detection of the full chessboard earlier. While the training set does not explicitly include images of chessboards under such extreme angles (otherwise, the network trained on the clean set would also have a similar performance), the lens distortions simulated in the full training set mean that MATE is able to better cope with large distortions, apparently perspective ones as much as lens distortions. This feature is of particular importance in multi-camera set-ups: it is far less likely that all of the imaging planes will be parallel, and hence the chessboard will be on non-zero angles to the various imaging planes.

### 3.4. Impact of the Training Set Size

**Figure 9:**
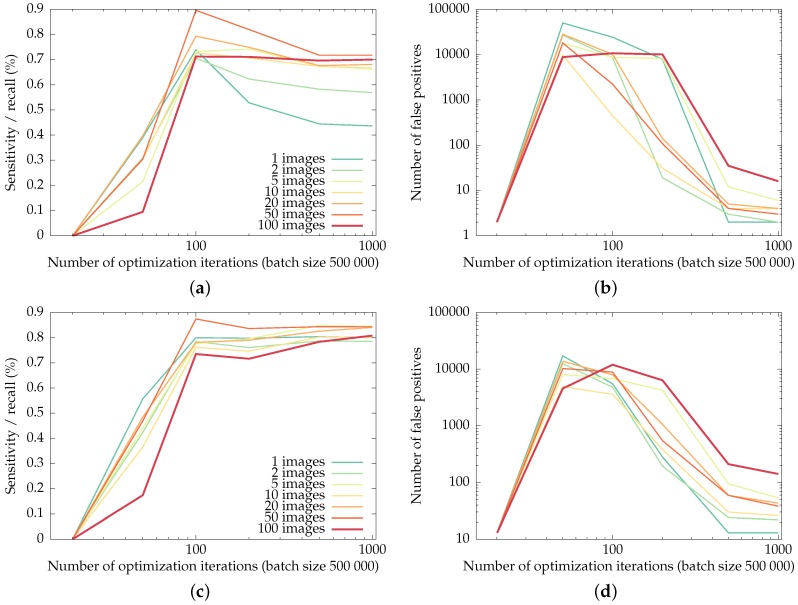
Recall of the checkerboard corners (the fraction of corners detected as such) and the number of false positives in the uEye and GoPro datasets for networks trained on fractions of the training set, as a function of the training iterations. Training was done in batches of 500000 backpropagations, i.e., an entire image at once. (**a**) and (**b**): recall and false positives for the uEye dataset, (**c**) and (**d**): recall and false positives for the GoPro dataset.

In order to test the impact of the training set size, we retrain the network using only a fraction of $P_{\mathrm{clean}}$, for a fixed number of backpropagations. For each evaluated training set size, ten random subsets of $P_{\mathrm{clean}}$ were evaluated; the averaged results are shown in Figure 9.

The general trend is that the networks trained on more input images allow for more false positives in order to boost recall of the checkerboard corners: for a low number of images, the networks tend to over-fit, resulting in less false positives and lower recall. The largest number of false positives for the most-trained networks in this experiment is reached by the network trained on the entire $P_{\mathrm{clean}}$, for the GoPro dataset: however, the 141 false positives only amount to roughly a single false positive per input image.

The overshoot visible in the recall curves illustrates the typical behavior of the optimization: initially, none of the checkerboard corners are detected. Only after the number of detections and false positives is in balance does the training phase start to weed out false positives.

### 3.5. Impact of the Spatial Support of the First Layer

**Figure 10:**
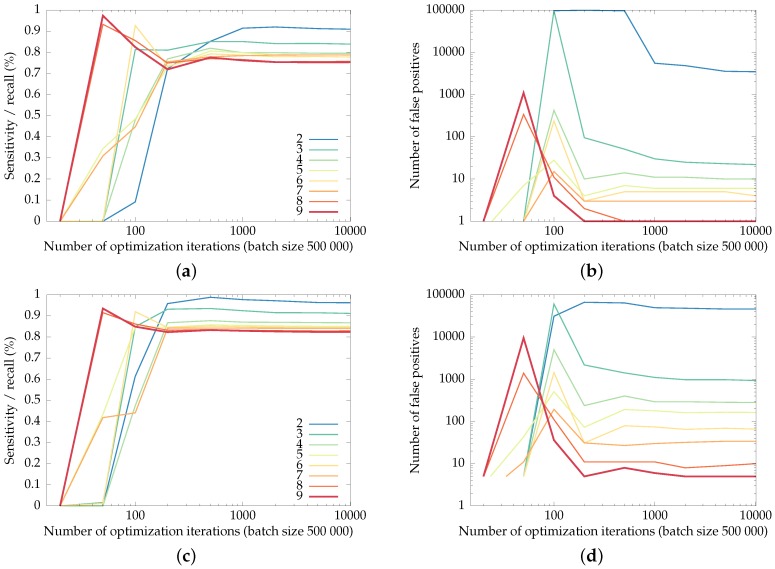
Recall of the checkerboard corners (the fraction of corners detected as such) and the number of false positives in the uEye and GoPro datasets for networks with varying spatial support radii, as a function of the training iterations. (**a**) and (**b**): recall and false positives for the uEye dataset, (**c**) and (**d**): recall and false positives for the GoPro dataset.

**Figure 11:**
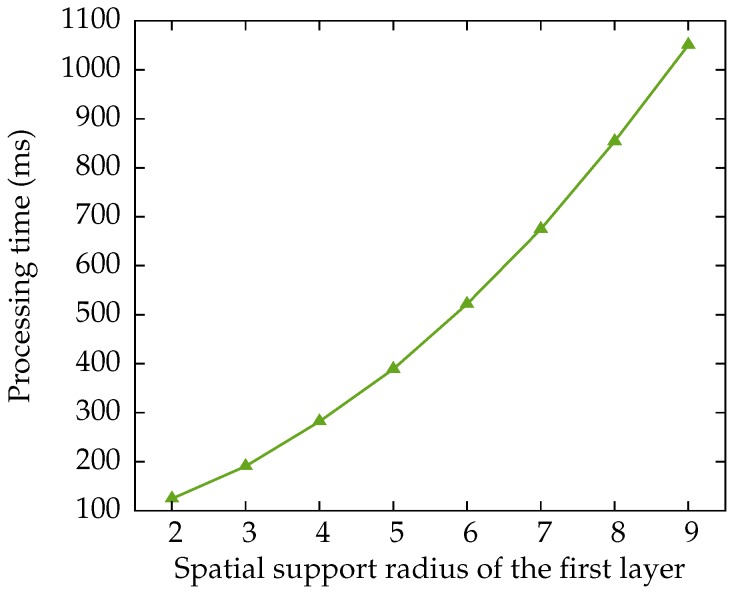
Average processing time required for the various spatial supports in the first layer, for an image in the uEye dataset. This time includes the application of the threshold, the non-maximum suppression and the creation of the list with detected pixel locations.

In this subsection, we explore the parameter space for the radius of the first convolutional layer. We train the network, with a varying spatial support, on $P_{\mathrm{full}}$ for 10,000 iterations of 500,000 backpropagations. The performance of the various networks, in terms of their recall and number of false positives, is shown in Figure 10. The required execution time for the various spatial support radii is shown in Figure 11.

Note that the training phase optimizes the number of relative mistakes—the percentage of missed checkerboard corners versus the percentage of background pixels falsely detected as checkerboard corners.

This balancing between recall and precision explains the trend in Figure 10: networks with a large spatial radius are better able to suppress false positives than networks with smaller supports, although they lose some recall of the checkerboard corners. Networks with small spatial supports (two and three pixels, mainly) result in an exorbitant number of false positives: up to several hundred false positives per frame for a spatial support of two pixels on the GoPro dataset.

We have chosen for a network with a spatial support radius of six pixels for the earlier comparisons, which allows us to compare more meaningfully with CheSS [20], which has the same spatial radius. The choice of spatial support will hence vary between applications, depending on the processing time·available.

**Figure 12:**
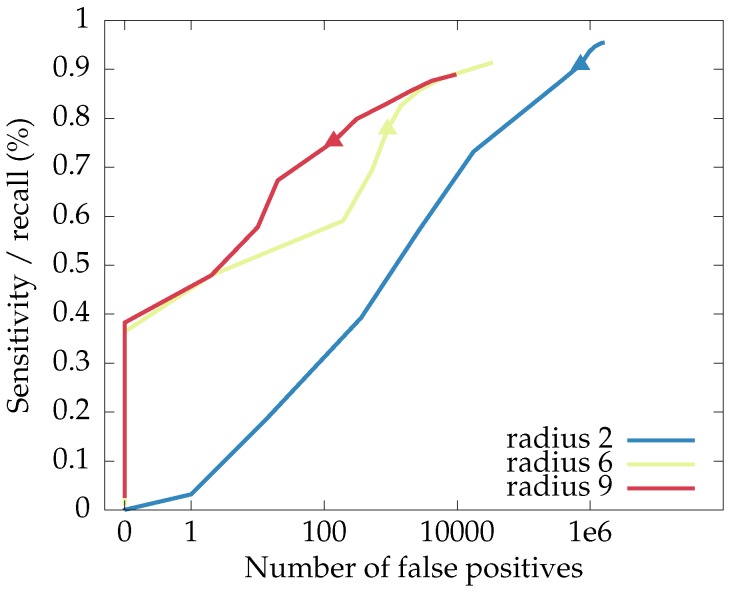
The curves for recall and number of false positives in the function of the threshold used for the response maps of the proposed neural network, with the point for a threshold of 0.5 highlighted. Higher-lying curves are better.

However, we remind the reader that the proposed neural network approach already has a method for trading between recall and false positives: the threshold applied to the response maps output by the network. Figure 12 shows the recall-false positives curves for networks with radii 2, 6 and 9, in the function of the applied threshold. From this plot, we conclude that, while the choice of threshold can be used to trade between recall and precision, (well-trained) networks with larger spatial supports will offer better performance: a higher precision-recall curve.

### 3.6. Application to a CMYK Hexboard

Due to the straightforwardness of training a neural network, attuning it to specific scenarios is much less labor intensive than designing hand-tailored features for each application. In this section, we briefly illustrate this property for our proposed detector.

To stamp down on false positives and use all information available from a consumer color camera, we have designed a CMYK (cyan, magenta, yellow and black) calibration plane. The CMYK colorspace was chosen because it is straightforward to print with consumer printers that print subtractively using CMYK ink or dust. Using a hex tiling, the corners are equivalent up to a 120 degree rotation and/or a reflection. As mentioned in Section 2, this multi-channel input is taken into account by adopting the formulation from Equation (4) as the first layer. The rest of the network remains the same.

**Figure 13:**
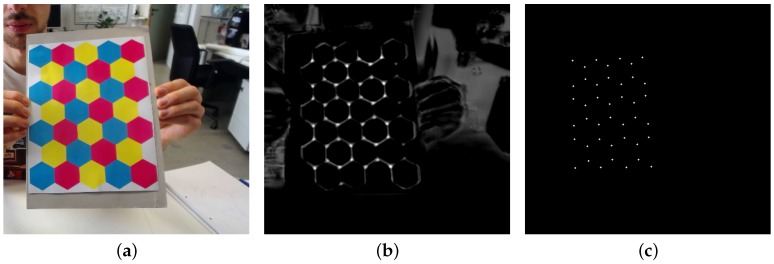
Result for the neural net trained on a CMYK hexboard input. The input image (**a**); the response map (**b**); and the detected calibration template corners (**c**).

We will not get into a discussion on the merits or disadvantages of a non-square calibration object in this paper; it serves as an illustration of the broadness of the proposed neural network architecture. After a training phase similar to the one outlined in Section 3.1, the network is able to detect the corners of the hexboard well, as illustrated in Figure 13.

## 4. Discussion and Conclusions

In this paper, we have presented a novel method to detect checkerboard corners. Motivated by the success of circular boundary methods, we propose a neural network that is a generalization of circular boundary methods on which several existing methods can be directly mapped. After a training phase, it is shown that the proposed technique performs at least as well as the state-of-the-art methods.

A robustly trained variant is able to detect checkerboard corners reliably in severely distorted scenarios. Notably, MATE is able to retain its performance even under scenarios with a large amount of noise. The generality of the neural network formulation is illustrated by training the network on an alternate calibration object, exploiting the color information present in nearly all consumer cameras. The neural network can be trained easily for new scenarios and applications, re-using the same network architecture.

## Figures and Tables

**Table 1:** Results on the training set $P_{\mathrm{clean}}$.

Method	Accuracy (px)	Complete Checkerboards	Missed Corners (%)	Double Detections (%)	False Positives	Time (ms)
MATE${}^{*}$	1.009	104/104	0.000	0.000	0	246
MATE	1.160	103/104	0.020	2.444	40	264
ChESS	1.094	104/104	0.000	0.280	10	212
ROCHADE	1.130	104/104	0.000	0.000	0	6458
OCamCalib	0.758	52/52	1.202	0.000	0	160

**Table 2:** Results on the training set $P_{\mathrm{full}}$.

Method	Accuracy (px)	Complete Checkerboards	Missed Corners (%)	Double Detections (%)	False Positives	Time (ms)
MATE${}^{*}$	0.810	66/104	1.162	0.020	0	242
MATE	0.999	81/104	0.62	4.147	40	246
ChESS	1.042	73/104	0.842	0.902	15	209
ROCHADE	0.423	38/104	54.899	0.000	0	6642
OCamCalib	1.084	52/52	30.967	0.000	1	243

**Table 3:** Results on the uEye dataset from [14].

Method	Accuracy (px)	Complete Checkerboards	Missed Corners (%)	Double Detections (%)	False Positives	Time (ms)
MATE${}^{*}$	0.886	181/206	3.497	0.009	12	531
MATE	1.009	186/206	3.065	0.809	492	529
ChESS	0.946	175/206	3.398	0.000	11	473
ROCHADE	1.510	186/206	2.895	0.000	1	6753
OCamCalib	0.319	206/206	0.000	0.000	0	261

**Table 4:** Results on the GoPro dataset from [14].

Method	Accuracy (px)	Complete Checkerboards	Missed Corners (%)	Double Detections (%)	False Positives	Time (ms)
MATE${}^{*}$	1.323	81/100	10.556	0.000	12	1209
MATE	0.835	86/100	4.556	4.556	389	1205
ChESS	1.389	80/100	5.481	0.222	56	1080
ROCHADE	1.807	80/100	5.593	0.000	3	6688
OCamCalib	0.458	100/100	0.537	0.000	0	533
